# Two-dimensional speckle tracking echocardiography in assessing the subclinical myocardial dysfunction in patients with gestational diabetes mellitus

**DOI:** 10.1186/s12947-022-00292-3

**Published:** 2022-08-09

**Authors:** Wei Li, Ziyao Li, Wei Liu, Peng Zhao, Guoying Che, Xudong Wang, Zhixin Di, Jiawei Tian, Litao Sun, Zhenzhen Wang

**Affiliations:** 1 Cardiovascular Center, Department of Ultrasound Medicine, Zhejiang Provincial People’s Hospital, Affiliated People’s Hospital of Hangzhou Medical College, Hangzhou, China; 2 Ultrasound Department, Second Affiliated Hospital of Harbin Medical University, Harbin, China

**Keywords:** Gestational diabetes mellitus, Speckle tracking echocardiography, LA strain, LA phasic function, Global longitudinal strain

## Abstract

**Background:**

Gestational diabetes mellitus (GDM) may increase the risk of cardiovascular disease and accompany asymptomatic deterioration of the myocardial function. This study aims to identify the subclinical impact of GDM on maternal left ventricular function by two-dimensional speckle tracking echocardiography (2D-STE).

**Methods:**

We prospectively recruited 47 women with GDM and 62 healthy pregnant women who underwent transthoracic echocardiography (TTE) at 24 to 28 weeks of pregnancy. GDM diagnosis agreed with the IADPSG criteria. TTE was performed according to the criteria of the American Society of Echocardiography. Conventional echocardiographic data and 2D-STE parameters were compared between the two groups.

**Results:**

Age, gestational weeks, heart rate, and conventional echocardiographic parameters had no difference between the two groups. The average LV global longitudinal strain (LV-GLS) of GDM patients was lower than controls (18.14 ± 2.53 *vs.* 22.36 ± 6.33, *p* < 0.001), and 31 patients (66%) in our study had an absolute LV-GLS less than 20%. The LA reservoir and conduit strain in patients with GDM were also significantly reduced (32.71 ± 6.64 *vs.* 38.00 ± 7.06, 20.41 ± 5.69 *vs.* 25.56 ± 5.73, *p* < 0.001). However, there was no significant difference in LA contractile function between the two groups. In multiple regression analysis, LV-GLS and LA conduit strain independently associated with GDM.

**Conclusions:**

2D-STE could detect the subclinical myocardial dysfunction more sensitively than conventional echocardiography, with LV-GLS and LA conduit strain as independent indicators of the GDM impact on maternal cardiac function during pregnancy.

## Introduction

Diabetes mellitus (DM) is a systemic metabolic disease that may lead to multiple organ dysfunction, among which cardiovascular impairment is relatively prominent [1, 2]. The number of diabetic patients worldwide is increasing rapidly, and it has threatened young people even pregnant women [3, 4]. Gestational diabetes mellitus (GDM) is a special entity, which refers to diabetes first diagnosed during pregnancy [5–7].

Patients with DM may be asymptomatic, with decreased myocardial diastolic function but preserved left ventricular ejection fraction (LVEF) [1, 8]. Two-dimensional speckle tracking echocardiography (2D-STE) has become a powerful tool to describe the subclinical deterioration of myocardial function in cardiovascular disease. However, it has not been widely introduced to GDM. We assume that the myocardial dysfunction already exists at the GDM diagnosis. Early detection of subclinical cardiovascular changes may be crucial for optimizing clinical management and preventing future cardiovascular events. Therefore, in this study, we performed 2D-STE to evaluate the LV diastolic and systolic function in patients with GDM and try to determine parameters that may identify the early impact of GDM on maternal myocardial function.

## Materials and methods

### Study population

The study was approved by the Ethics Committee of Harbin Medical University. From October 2020 to January 2021, we recruited 124 consecutive Chinese women with a singleton pregnancy who underwent comprehensive transthoracic echocardiography (TTE) assessment. All the patients have signed the informed consent before examination. Fifteen patients were excluded due to their poor acoustic window, and finally 47 GDM patients and 62 healthy pregnant women were included in the study.

The International Association of Diabetes and Pregnancy Study Groups (IADPSG) defined GDM as any degree of low glucose tolerance first diagnosed during pregnancy [9]. The diagnosis of GDM was made by performing the 75 g oral glucose tolerance test (75 g OGTT) between 24 and 28 weeks. The diagnose criteria includes fasting plasma glucose (FPG) ≥ 5.1 mmol/l (92 mg/dL), 1-h plasma glucose ≥ 10.0 mmol/l (180 mg/dL), and 2-h plasma glucose ≥ 8.5 mmol/l (153 mg/dL). In this study, GDM patients should have normal LVEF (≥ 54%). Demographic and clinical data were routinely recorded before their recruitment. The patients have no history of relevant cardiovascular diseases or other metabolic diseases and deny smoking or drinking habit. The method of conception was natural.

### Clinical information

The age, body mass index (BMI), blood pressure (BP), heart rate (HR), gestational weeks, and blood glucose level of the study population were queried at their enrollment. BP was measured three times and averaged after at least ten minutes of rest. BP was measured in a silent room 5 to 10 min before echocardiography with an aneroid sphygmomanometer twice in a seated position, with the right arm at the level of the heart, after 5 min of rest.

### Ultrasound protocol

#### Conventional echocardiography

Echocardiography was performed by two senior sonographers (Ziyao Li and Wei Li) on GE Vivid E9 and E95 (GE Medical Systems, Milwaukee, WI, USA) with an M5S probe (2.5 ~ 4.0 MHz). All data were averaged from three consecutive cardiac cycles. Patients with poor image quality were excluded before recruitment. Images were recorded and studied according to the recommendations of the American Society of Echocardiography [10].

In the parasternal long-axis view, LV end-diastolic diameter (LVEDd), interventricular septum (IVS) thickness, posterior wall thickness (PWT), and LV end-systolic diameter (LVESd) were measured by M-mode echocardiography. LV mass (LVM) was calculated by using the Devereux formula [11]: LVM = 0.8 × {1.04 × [(LVEDd + IVS + PWT)^3^–LVEDd^3^]} + 0.6g. Relative wall thickness (RWT) was calculated using the formula RWT = 2 × (PWT/LVEDd). LVEF and LA volume (LAV) were measured using the biplane Simpson method. LVM, LAV, and stroke volume (SV) were indexed for body surface area (BSA) to get LV mass index (LVMI), LA volume index (LAVI), and stroke volume index (SVI), respectively. In the apical four-chamber view, pulse Doppler and tissue Doppler were performed to measure early diastolic mitral inflow velocity (E), and early diastolic annular velocity (e′). And mean e′ was the averaged velocity of the septal and lateral mitral annulus [12].

### Two-dimensional speckle tracking echocardiography

LV global longitudinal strain (LV-GLS) and LA phasic strain were analyzed offline using EchoPAC software (version 203, GE Healthcare, Horten, Norway). Allow the patient to hold their breath to get ultimate images of three consecutive cardiac cycles at a frame rate ≥ 60 frames per second. The 2D-STE measurements were performed by two physicians in a double-blinded manner for intraclass correlation coefficients (ICC) testing.

To measure LV-GLS, 2D-STE was performed by tracing the LV endocardial boundary in the apical three-chamber, four-chamber, and two-chamber views [13]. We use the apical three-chamber view to identify the aortic valve closure and then mark the mitral annulus points and apex in each apical view. The software can track the endocardial border and automatically generate six segments of longitudinal strain from each apical view separately, and then LV-GLS is averaged from all those 18 segments.

The biplane (4-chamber and 2-chamber) views were accepted for LA strain evaluation, according to the consensus from the European Association of Cardiovascular Imaging (EACVI)/American Society of Echocardiography (ASE)/Industry Task Force [14]. When tracing the LA endocardial border, the atrial appendage and pulmonary veins were eliminated. Then six segmental LA longitudinal strain curves were automatically presented by the software. An R-R gating protocol was applied to get the LA phasic strain, which including reservoir strain (LA-Sr), conduit strain (LA-Scd), and contractile strain (LA-Sct) [15].

### Statistical analysis

Continuous variables were expressed as mean ± standard deviation (SD) and compared by the student *t*-test. We firstly performed the univariate logistic regression to assess the crude correlations between clinical/echocardiographic characteristics and GDM. Variables with a *p-*value less than 0.05 in univariate regression entered the multivariate models, and a forward “likelihood ratio” selection approach was applied to identify parameters that were independently associated with GDM. The current study conducted two multivariate models which separately included either LV-GLS or LA phasic strain, to better identify their associations with GDM. ICC was examined by the Bland–Altman plot. We used SPSS version 25.0 (IBM Corporation, Armonk, NY) statistical software. A *p*-value less than 0.05 was considered statistically significant.

## Results

### Clinical characteristics

Table 1 shows the clinical characteristics of the study population. There were no significant differences between the two groups regarding age, gestation-week, and heart rate (all *p* > 0.05). Compared with the control group, GDM had increased BMI (27.87 ± 4.11 *vs.* 24.76 ± 2.92 kg/m^2^, *p* < 0.001), higher SBP (117.81 ± 8.10 *vs.*113.73 ± 9.17 mmHg, *p* = 0.017) and DBP (78.04 ± 5.74 *vs.*75.08 ± 7.72 mmHg, *p* = 0.029). Based on the GDM level, only diet treatment was recommended clinically, no oral hypoglycemic drugs or insulin therapy were initiated.

**Table 1:** Clinical characteristics of the study population

Variables	Controls	GDM	*p-value*
Age (years)	30.74 ± 4.55	30.74 ± 4.67	0.998
Gestation week	27.50 ± 3.24	28.82 ± 4.95	0.096
BMI (kg/m^2^)	24.76 ± 2.92	27.87 ± 4.11	< 0.001
SBP (mmHg)	113.73 ± 9.17	117.81 ± 8.10	0.017
DBP (mmHg)	75.08 ± 7.72	78.04 ± 5.74	0.029
HR (bpm)	90.11 ± 11.54	92.68 ± 10.61	0.236
FPG (mmol/l)	4.26 ± 0.27	5.55 ± 1.65	< 0.001

Data were presented as mean ± SD and compared by the student *t*-test

*GDM* Gestational diabetes mellitus, *BMI* Body mass index, *SBP* Systolic blood pressure, *DBP* Diastolic blood pressure, *HR* Heart rate, *FPG* Fasting plasma glucose

### Conventional echocardiography

Table 2 shows the conventional echocardiographic parameters of the two groups. Compared with control, GDM had bigger IVS, LVPW, RWT, and LVMI (all *p* < 0.001). LVEF was preserved in GDM and had no statistical difference with control. The mean e' velocity of mitral annulus was lower in GDM than control (13.24 ± 2.34 *vs.* 14.67 ± 2.17 cm/s, *p* = 0.002). However, there was no difference regarding the peak mitral inflow velocities (E and A), E/A ratio, or mean E/e'.

**Table 2:** Conventional echocardiographic parameters of the study population

Variables	Controls	GDM	*p-value*
IVS (mm)	8.40 ± 0.93	9.46 ± 1.07	< 0.001
LVPW (mm)	8.57 ± 0.90	9.61 ± 0.99	< 0.001
LVEDd (mm)	44.01 ± 2.58	44.88 ± 3.09	0.112
LVESd (mm)	22.90 ± 2.92	23.89 ± 2.87	0.080
RWT	0.39 ± 0.04	0.43 ± 0.05	< 0.001
LVMI (g/m^2^)	69.65 ± 12.92	79.86 ± 14.77	< 0.001
LVEF (%)	68.08 ± 5.59	66.26 ± 6.73	0.135
SVI (ml/m^2^)	37.66 ± 7.35	35.24 ± 6.81	0.079
LAVI (ml/m^2^)	24.94 ± 6.12	23.80 ± 5.77	0.322
E velocity (cm/s)	94.16 ± 14.89	90.76 ± 16.72	0.277
A velocity (cm/s)	63.53 ± 13.56	67.09 ± 15.94	0.226
E/A	1.54 ± 0.35	1.54 ± 0.92	0.975
Mean e' (cm/s)	14.67 ± 2.17	13.24 ± 2.34	0.002
Mean E/e'	6.50 ± 1.17	7.09 ± 1.77	0.053

*GDM* Gestational diabetes mellitus, *IVS* Interventricular septum, *LVPW* Left ventricular posterior wall, *LVEDd* Left ventricular end-diastolic diameter, *LVESd* Left ventricular end-systolic diameter, *RWT* Relative wall thickness, *LVMI* Left ventricular mass index, *LVEF* Left ventricular ejection fraction, *SVI* Stroke volume index, *LAVI* Left atrial volume index

### Two-dimensional speckle tracking echocardiography

**Fig. 1:**
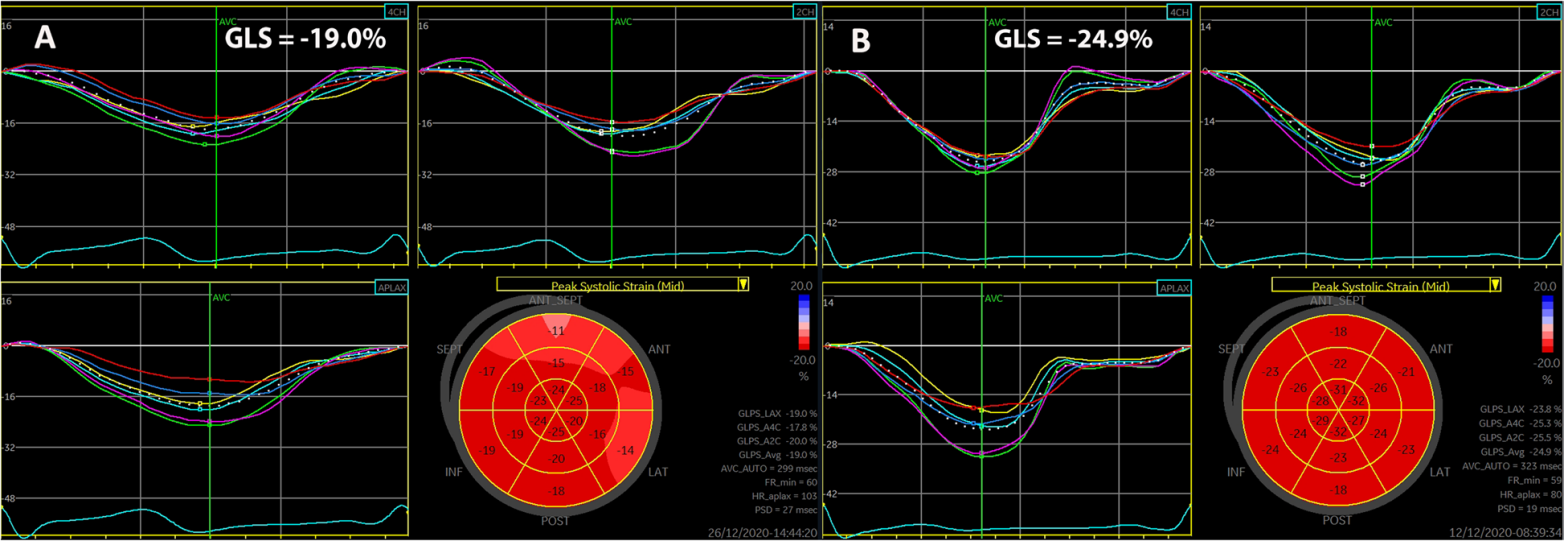
Offline analysis of 2D-STE depicts LV-GLS of three apical views from a GDM woman (**A**) and a healthy control (**B**). Bull’s eye view shows segmental peak systolic strain values and the averaged LV-GLS. LV-GLS, LV global longitudinal strain

**Fig. 2:**
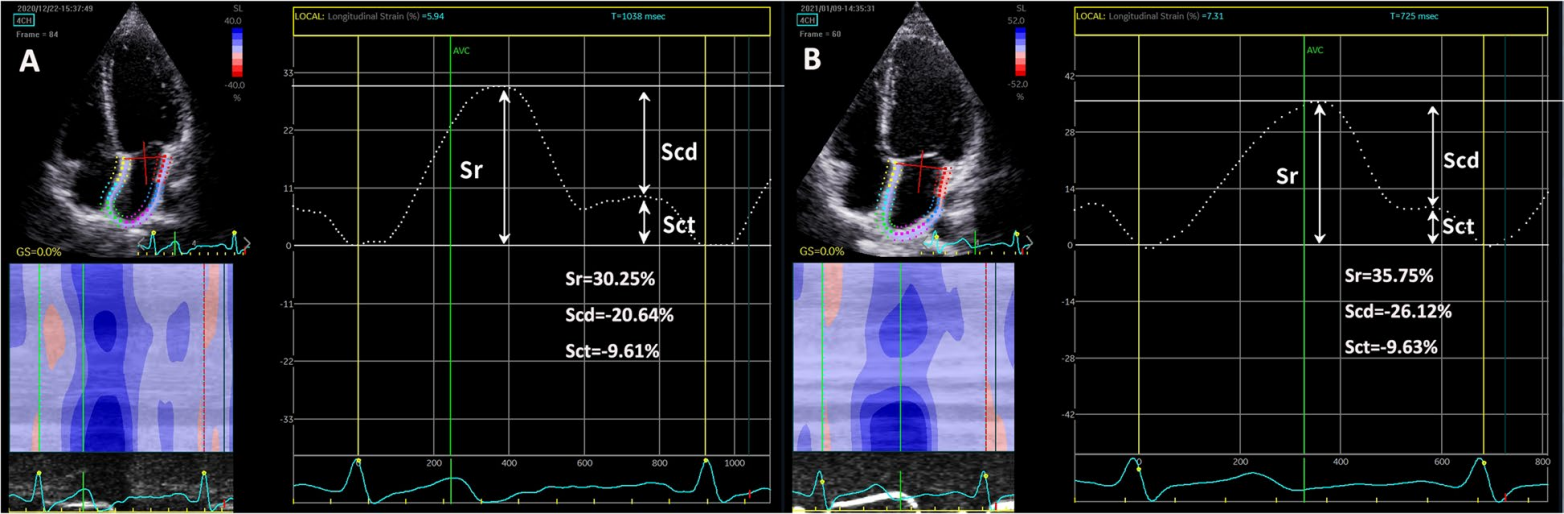
Four-chamber views present phasic LA strain of a GDM woman (**A**) and a healthy control (**B**). Sr, LA reservoir strain; Scd, LA conduit strain; Sct, LA contractile strain

LV-GLS and LA phasic strain of the study population are depicted in Table 3. The amplitude of LV-GLS in GDM patients was significantly lower than normal pregnant women (18.14 ± 2.53 *vs.* 22.36 ± 6.33, *p* < 0.001) (Fig. 1), and 31 patients (66%) in our study had an absolute LV GLS less than 20%. As for the absolute value of LA phasic strain, LA-Sr and LA-Scd were significantly lower than the control group (32.71 ± 6.64 *vs.* 38.00 ± 7.06, and 20.41 ± 5.69 *vs.* 25.56 ± 5.73, respectively, *p* < 0.001). However, LA-Sct had no difference between the two groups (*p* > 0.05) (Fig. 2).

**Table 3:** LV-GLS and LA phasic strain of the study population

Variables	Controls	GDM	*p-value*
LV-GLS (%)	23.09 ± 2.49	18.14 ± 2.53	< 0.001
LA-Sr (%)	38.00 ± 7.06	32.71 ± 6.64	< 0.001
LA-Scd (%)	25.56 ± 5.73	20.41 ± 5.69	< 0.001
LA-Sct (%)	14.80 ± 3.98	14.01 ± 3.74	0.298

*GDM* Gestational diabetes mellitus, *LV* Left ventricular, *LA* Left atrial, *LV-GLS* Left ventricular global longitudinal strain, *LA-Sr* Left atrial reservoir strain, *LA-Scd* Left atrial conduit strain, *LA-Sct* Left atrial contractile strain

### Regression analyses

In Table 4, univariate logistic regression analysis shows that BMI, SBP, DBP, RWT, LVMI, mean e', LV-GLS, LA-Sr, and LA-Scd were associated with GDM. In the multivariate model that focused on LV-GLS, LV-GLS (OR, 0.439; 95% CI, 0.320–0.603; *p* < 0.001) was independently associated with GDM. In another model that mainly involved LA phasic strain, LA-Scd showed a good independent association with GDM (OR, 0.874; 95% CI, 0.802–0.952; *p* = 0.002) (Table 5).

**Table 4:** Univariate logistic regression analysis of GDM associated parameters

Variables	OR (95% CI)	*p-value*
Age (years)	1.000 (0.920–1.087)	0.998
Gestation week	1.084 (0.985–1.194)	0.100
BMI (kg/m^2^)	1.289 (1.135–1.465)	< 0.001
SBP (mmHg)	1.057 (1.008–1.108)	0.021
DBP (mmHg)	1.066 (1.005–1.130)	0.033
HR (bpm)	1.021 (0.986–1.057)	0.235
RWT	3.003 (1.329–6.787)	0.008
LVMI (g/m^2^)	1.054 (1.023–1.086)	0.001
LVEF (%)	0.952 (0.893–1.014)	0.126
SVI (ml/m^2^)	0.952 (0.901–1.007)	0.084
LAVI (ml/m^2^)	0.968 (0.907–1.033)	0.324
E (cm/s)	0.986 (0.962–1.011)	0.266
A (cm/s)	1.017 (0.990–1.044)	0.214
E/A	1.011 (0.563–1.815)	0.971
Mean e' (cm/s)	0.749 (0.620–0.904)	0.003
Mean E/e'	1.278 (0.993–1.646)	0.057
LV-GLS (%)	0.461 (0.349–0.608)	< 0.001
LA-Sr (%)	0.895 (0.842–0.952)	< 0.001
LA-Scd (%)	0.855 (0.791–0.924)	< 0.001
LA-Sct (%)	0.948 (0.858–1.048)	0.300

*OR* Odds ratio, *CI* Confidence interval, Other abbreviations were as shown in Tables 1, 2, and 3

**Table 5:** Multivariate regression analysis for identifying variables independently associated with GDM

Variables	OR(95% CI)	*p-value*
Model 1^a^		
LV-GLS (%)	0.439 (0.320–0.603)	< 0.001
Mean e' (cm/s)	0.695 (0.512–0.943)	0.020
Model 2^b^		
LA-Scd (%)	0.874 (0.802–0.952)	0.002
BMI (kg/m^2^)	1.173 (1.025–1.342)	0.020
LVMI (g/m^2^)	1.041 (1.005–1.078)	0.023

^a^Adjusted for BP, BMI, RWT and LVMI; ^b^Adjusted for BP, RWT, mean e' and LA-Sr

*OR* Odds ratio, *CI* Confidence interval, Other abbreviations were as shown in Tables 1, 2, and 3

### Reproducibility of strain measurements

**Fig. 3:**
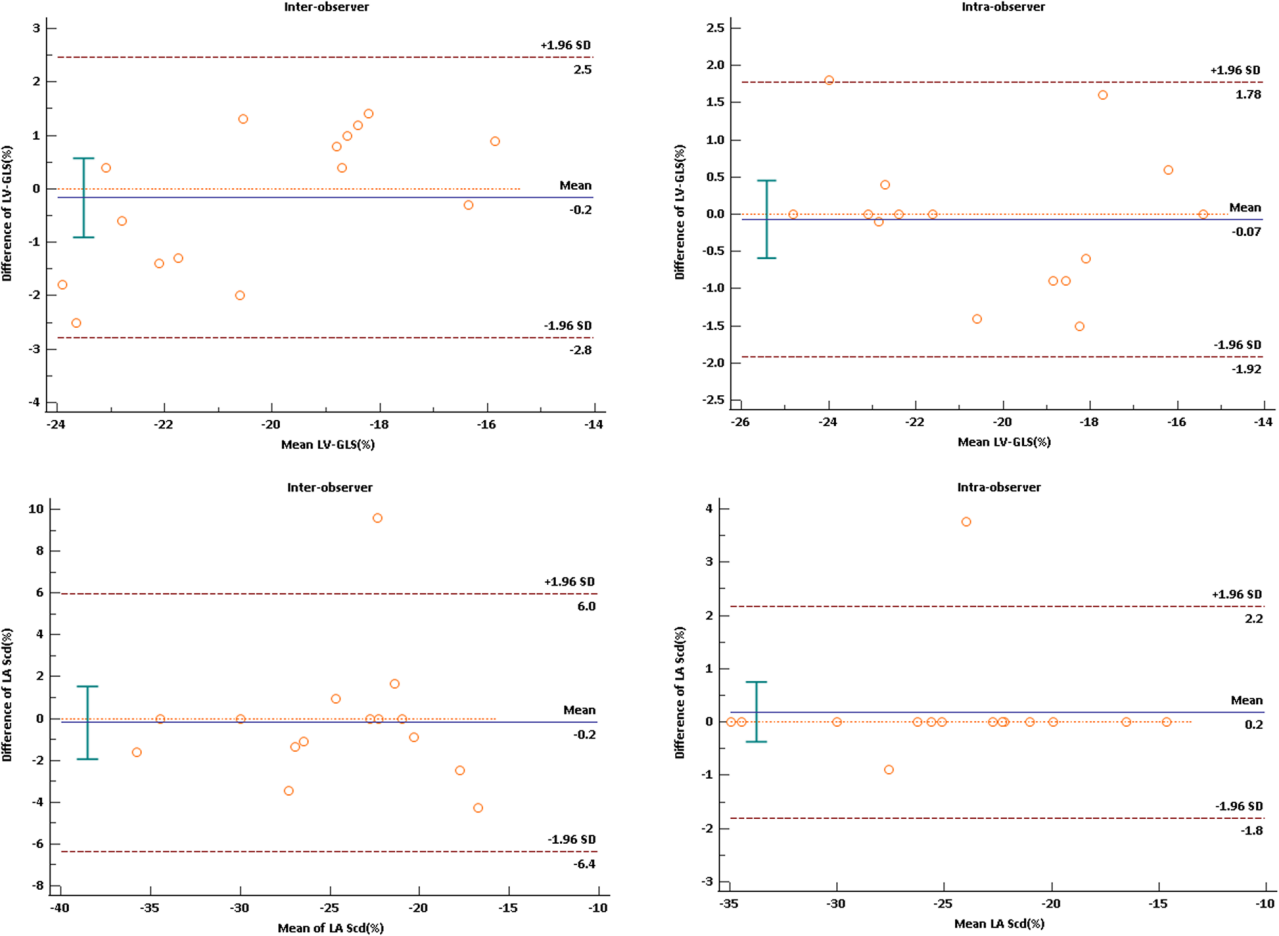
Bland–Altman plot shows the inter-observer and intra-observer agreements of strain measurements (LV-GLS, and LA-Scd)

To assess the reproducibility of strain measurements, we randomly selected 15 patients from the study population for the ICC test. There was good reproducibility between inter-observer and intra-observer measurements (Fig. 3, Table 6).

**Table 6:** Intraclass correlation coefficients tests of strain measurements

Variables	Intra-observer ICC	Inter-observer ICC
LV-GLS (%)	0.950	0.908
LA-Sr (%)	0.982	0.920
LA-Scd (%)	0.984	0.811
LA-Sct (%)	0.929	0.865

*ICC* Intraclass correlation coefficients, Other abbreviations were as shown in Table 3

## Discussion

GDM is one of the most common complications of pregnancy [16]. Given that DM is a risk factor for future cardiovascular events [17–19], the impact of GDM on maternal cardiac function changes could not be ignored. Aiming to early detecting the myocardial dysfunction in newly diagnosed GDM women, we compared the 2D-STE with conventional echocardiography during their 24 ~ 28 weeks of gestation. The main findings of the study were as follows: [1] GDM preserved LV systolic and diastolic function by conventional echocardiography and had no difference with control; [2] LV-GLS provides early information of LV systolic myocardial deformation in GDM; [3] LA conduit strain may be the prominent phasic parameter to early identify LV diastolic dysfunction in GDM.

Owing to the hemodynamic changes during normal pregnancy [20, 21], physiological remodeling of the myocardium may occur [22, 23]. In GDM, hyperglycemia and insulin resistance may lead to the disruption of Ca^2+^ balance, the accumulation of advanced glycation end products (AGEs), and the increase of oxidative stress and inflammation. They may trigger extracellular matrix accumulation, cardiomyocyte apoptosis, and myocardial fibrosis. Eventually, the left ventricle will develop centripetal hypertrophy and diastolic dysfunction [24–26]. As expected, we found that GDM women had thicker myocardium, higher RWT, and increased LVMI than normal pregnant women, indicating the myocardial remodeling may accompany GDM progression. Similar to the findings by Merra et al. [27], we found LV-GLS of GDM was lower than that of controls despite their normal LVEF. In the setting of GDM may have an association with obesity [28], we also found an increased BMI in our GDM patients. Of note, after adjusting confounders that include BMI, LV-GLS could remain its independent association with GDM, indicating LV-GLS may serve as an indicator of subclinical systolic dysfunction of GDM.

On the other hand, LV diastolic function may also deteriorate in GDM patients. Among all the conventional echocardiography biomarkers of LV diastolic function, only the mean e' was independent associated with GDM. Although LA remodeling is considered a signal of LV diastolic functional changes [29], LAVI did not present a significant difference between GDM and controls. Considering the atrioventricular coupling, we also conducted LA phasic (reservoir, conduit, and contractile) strain analysis in GDM women. During LV systole and isovolumic relaxation, LA performs as a reservoir, receiving blood from pulmonary veins. The conduit phase is modulated especially by LV diastolic properties (relaxation and early diastolic pressure). LA contractile performance, also called booster-pump function, is modulated by LV compliance, LV end-diastolic pressure, and LA intrinsic contractility [29–33]. LA reservoir and conduit strain have been reported to correlate with LV filling pressure [34] and may gradually decrease even in mild LV diastolic dysfunction progression [35]. We found LA-Sr and LA-Scd were significantly lower than controls, and LA-Scd had an independent association with GDM, which superior to mean e'. Such findings suggest firstly that 2D-STE is a potential tool to recognize LA functional changes in GDM, and secondly, the LV relaxation may be impaired and LV filling pressure may increase in GDM women. Clinical management should be concerned before further deterioration of LV diastolic function happens. Furthermore, the results from the ICC test support the good performance and clinical role of strain assessment in myocardial function.

### Limitations

The current study reveals the ability of 2D-STE to distinguish the difference in myocardial function between newly diagnosed GDM and healthy pregnant women with preserved LVEF. However, there are intrinsic limitations of the current study. Firstly, the sample size of the study was small and from a single center. Secondly, we are lacking the information regarding the normal threshold of LV GLS and LA strains in pregnant women. Thirdly, we currently do not have either short-term or long-term follow-up information of GDM patients regarding the subclinical myocardial deformation impact on future CVD events, further observations are still needed regarding the cardiovascular outcomes in patients with GDM.

## Conclusion

This is a preliminary study on the performance of 2D-STE in GDM, and due to the limited number of patients and lack of follow-up information, the results need to be confirmed by larger studies. However, we have elucidated that 2D-STE may serve as a powerful indicator of transient myocardial deterioration in GDM.

## Data Availability

Data and materials could be retrieved from the echo workstation of our institution if needed.
